# Supplementary material for: Impact of Psychopathology and Gut Microbiota on Disease Progression in Ulcerative Colitis: A Five-Year Follow-Up Study
Source: Microorganisms. 2025 May 25;13(6):1208. doi: 10.3390/microorganisms13061208 (PMC12195093; doi:10.3390/microorganisms13061208)
Supplement: Supplementary file 1 [file microorganisms-13-01208-s001.zip › microorganisms-3589379-supplementary.pdf]

			Mean ± SD
Clinical scales	**Hs (Hypochondriasis)**	*Abnormal, psychoneurotic concern over one's health. General malaise, pain, weakness, exhaustion, and illness, stomach difficulties, difficulty breathing, poor visual perception and other sensory disorders, cough, difficulty sleeping, dizziness, and body numbness are all mentioned.*	66.71 ± 11.30
	**D (Depression)**	*The scale reveals a clinical picture of a usually "negative" psychic structure: the subject reports feeling low in spirits, without hope for the future, dissatisfied with life, and having a gloomy mood. The items' content is obviously associated with depression, low self-esteem, loss of interest, and feelings of indifference.*	58.54 ± 11.32
	**Hy (Hysteria)**	*Somatic complaints, denial of psychiatric disorders, extroversion, or comfort in social relationships are all described in the items.*	57.69 ± 10.77
	**Pd (Psychopathic Deviate)**	*The overall score is closely connected with exterior actions that indicate aggressive, manipulative, and impulsive behavioral or family difficulties.*	54.37 ± 11.62
	**MF (Masculinity/Femininity)**	*The items cover interests and profession choices, with a small number reflecting psychological issues or symptoms. Occupational options are either stereotypically feminine (for example, librarian, nurse, flower artist) or masculine (for example, soldier, sports journalist, forest ranger).*	51.11 ± 13.73
	**Pa (Paranoia)**	*It assesses behavioral features associated with paranoid-type disorders, such as suspicion, distrust, fixed thoughts, excessive interpersonal sensitivity, rigid thinking, and critical attitude.*	51.91 ± 11.87
	**Pt (Psychasthenia)**	*It was originally designed to assess a psychological disorder (psychasthenia), which we now refer to as anxiety disorders with obsessive-compulsive features. It includes items that evaluate anxiety or overall maladjustment.*	59.83 ± 10.56
	**Sc (Schizophrenia)**	*High scores on this scale reflect a wide range of diagnostic possibilities, as significant elevations can occur in subjects with a variety of disorders, including schizophrenics, chronic psychiatric patients with emotional disorders, people with organic brain disorders, severely disturbed personalities, normal subjects with serious sensory system damage, non- conformists, rebels, and "counterculture" (for example, hippies in the 1960s).*	57.97 ± 9.56
	**Ma (Hypomania)**	*It assesses manic or hypomanic behavior, which is characterized by feelings of euphoria, aggression, and hyperactivity.*	57.77 ± 11.02
	**Si (Social Introversion)**	*The items' content addresses social discomfort, inferiority, poor sociability, interpersonal sensitivity, distrust.*	53.00 ± 11.40
Content scales	**ANX (Anxiety)**	*Detects widespread anxiety symptoms (tension, physical issues such as tachycardia and shortness of breath, sleep difficulties, excessive worrying, and concentration difficulties).*	60.60 ± 9.18
	**FRS (Fears)**	*Detects the presence of specific fears or phobias associated with various circumstances or things (blood, heights, money, animals such as snakes, mice, or spiders, leaving the house, fire, storms, and natural disasters, water, darkness, being in confined spaces, and dirt).*	59.60 ± 10.12
	**OBS (Obsessiveness)**	*Detects ruminative and compulsive thinking cognitive processes.*	59.77 ± 11.12
	**DEP (Depression)**	*Determine the presence of symptomatic depression (depressive-type thoughts, emotions of melancholy and uncertainty about one's future, loss of interest in one's life, increased emotional lability, feelings of emptiness, suicidal thoughts, wish for death).*	58.94 ± 9.81
	**HEA (Health Concerns)**	*Reports medical symptoms and complaints. Contains items that refer to a variety of physical symptoms that affect different parts of the body, such as gastrointestinal symptoms (constipation, nausea and vomiting, stomach upset), neurological problems (seizures, dizziness, fainting, paralysis), sensory problems (poor hearing or vision), cardiovascular symptoms (chest or heart pain), dermatological problems, pain (headache, neck pain), and breathing difficulties (cough, hay fever, asthma).*	65.91 ± 10.97
	**BIZ (Bizarre Mentation)**	*Detects severe symptoms of thought disorders (auditory, visual, or olfactory hallucinations, weird and peculiar thoughts, paranoid ideation, a sense of having a specific task to complete, or exceptional powers).*	55.63 ± 9.53
	**ANG (Anger)**	*Examine the loss of control that occurs when rage is expressed.*	56.23 ± 9.32
	**CYN (Cynicism)**	*This scale assesses cynical ideas and misanthropic attitudes.*	58.46 ± 8.93
	**ASP (Antisocial Practices)**	*It assesses antisocial personality traits (misanthropic sentiments similar to those indicated by high CYN scale scores).*	54.46 ± 9.41
	**TPA (Type A Behaviouor)**	*They identify an aggressive, hostile, and competitive personality style.*	55.37 ± 8.41
	**LSE (Low Self Esteem)**	*It identifies negative self-perception (a tendency to represent oneself negatively and have a low opinion of oneself).*	54.34 ± 8.47
	**SOD (Social Discomfort)**	*Assess social discomfort (the tendency to feel uncomfortable when around others and the urge to be alone).*	52.74 ± 9.82
	**FAM (Family Problems)**	*It focuses on family relationship issues (significant family discomfort, family that lacks affection, quarrelsome and unpleasant, hostility towards members of their family).*	56.86 ± 9.29
	**WRK (Work Interference)**	*Identifies difficulties and negative attitudes related to work or goal pursuit.*	60.40 ± 11.80
	**TRT (Negative Treatment Indicators)**	*Focuses on attitudes or difficulties experienced when seeking treatment or changing behavior (e.g., negative attitudes toward doctors and mental health therapies).*	59.60 ± 11.07

Supplementary Table S1. Description of MMPI-2 Clinical and Content Scales Used in the Study. This table provides an overview of the Minnesota Multiphasic Personality Inventory-2 (MMPI-2) scales and subscales analyzed in the present study.

The first section includes the 10 standard clinical scales, which assess core dimensions of adult psychopathology, while the second section includes the 15 content scales, which offer a more specific

evaluation of emotional, cognitive, and behavioral traits. Each scale is briefly described to facilitate understanding of its psychological significance and relevance to the study outcomes. For further details on the MMPI-2 scoring system and validated interpretive thresholds, including the clinical significance of elevated T-scores (e.g., T ≥ 65), see: Drayton, M. (2009). *The Minnesota Multiphasic Personality Inventory-2 (MMPI-2)*. Occupational Medicine (London), 59(2), 135–136. https://doi.org/10.1093/occmed/kqn182; Friedman, A. F., Bolinskey, P. K., Levak, R. W., & Nichols, D. S. (2014). *Psychological Assessment with the MMPI-2 / MMPI-2-RF* (1st ed.). Routledge. https://doi.org/10.4324/9780203119556. These sources provide comprehensive guidance on how to interpret the psychological meaning and diagnostic implications of individual scale scores.

For interpretative purposes, MMPI-2 raw scores are converted into standardized T-scores (mean = 50, SD = 10). According to established clinical guidelines, a T-score of **≥ 65** is typically considered clinically significant, indicating the potential presence of underlying psychological distress or dysfunction warranting further evaluation. Scores in the 65–74 range suggest moderate clinical concern, while scores of ≥ 75 may reflect severe or acute psychopathological states (e.g., psychosis or marked personality dysfunction). In the present dataset, two scales exceeded the clinical threshold (T ≥ 65): Hs (Hypochondriasis) in the Clinical Scales section, and HEA (Health Concerns) in the Content Scales section.

Note: SD (Standard Deviation).

Given the presence of extreme odds ratios in the logistic regression models, we provide here additional diagnostic outputs to ensure transparency and analytical robustness.

Model Fit Statistics

- **Omnibus Test of Model Coefficients:** $\chi^2(11) = 12.506$, $p = 0.327$
- **-2 Log Likelihood:** 32.498
- **Cox & Snell R^2:** 0.300
- **Nagelkerke R^2:** 0.415
- **Hosmer-Lemeshow Goodness-of-Fit Test:** $\chi^2(7) = 5.146$, $p = 0.642$

These results indicate an acceptable overall model fit, with no statistically significant lack of fit based on the Hosmer-Lemeshow test.

Regression Coefficients and Odds Ratios

A complete summary of the logistic regression coefficients (B), standard errors (S.E.), Wald statistics, and corresponding odds ratios [Exp(B)] with 95% confidence intervals is provided. Several predictors yielded extremely large or implausible odds ratios (e.g., Exp(B) > 10^90 or Exp(B) = 0.000), highlighting potential issues with model convergence and sparse data bias (**Table 1**).

Variable	B	S.E.	Wald	dl	*p*-value	Exp(B)	95% C.I. Lower	95% C.I. Upper
Age	0.017	0.032	0.264	1	0.608	1.017	0.954	1.083
Gender	-1.931	1.222	2.495	1	0.114	0.145	0.013	1.592
Actinobacteria	65.549	78.216	0.702	1	0.402	2.935E+28	0.000	1.109E+95
Bacteroidetes	89.443	80.351	1.239	1	0.266	6.990E+38	0.000	1.737E+107
Firmicutes	66.386	77.973	0.725	1	0.395	6.778E+28	0.000	1.591E+95
Proteobacteria	69.288	78.513	0.779	1	0.378	1.234E+30	0.000	8.345E+96
Verrucomicrobia	53.094	82.371	0.415	1	0.519	1.144E+23	0.000	1.488E+93
TM7	128.620	179.992	0.511	1	0.475	7.228E+55	0.000	1.171E+209
Euryarchaeota	-773.400	714.102	1.173	1	0.279	0.000	0.000	9.147E+271
Fusobacteria	-10584.86	4828.92	4.805	1	0.028	0.000	0.000	0.000
Tenericutes	2059.752	1818.77	1.283	1	0.257	0.000	0.000	0.000
Intercept	-67.294	77.971	0.745	1	0.388	0.000	0.000	0.000

Table 1. Dependent variable: "failure of three or more biologics". *Note:* B (Regression coefficient), Exp(B) (Exponentiated coefficient), df (Degrees of freedom), S.E.: Standard error of the regression coefficient B), Wald (Wald chi-square statistic).

Multicollinearity Assessment

We provide the full **correlation matrix** of independent variables included in the model. Very strong correlations were observed between several microbial phyla (e.g., Actinobacteria, Firmicutes, Proteobacteria), with coefficients nearing ±1.000, indicating substantial multicollinearity, which likely contributed to numerical instability and inflated coefficient estimates (**Table 2**).

	Intercept	Age	Gender	Actinobacteria	Bacteroidetes	Firmicutes	Proteobacteria	Verrucomicrobia	TM7	Euryarchaeota	Fusobacteria	Tenericutes
**Intercept**	1.000	0.001	0.106	-0.998	-0.991	-1.000	-0.999	-0.945	-0.704	0.012	0.123	-0.101
**Age**	0.001	1.000	-0.186	-0.026	0.010	-0.020	-0.011	-0.106	-0.005	-0.070	-0.099	0.079
**Gender**	0.106	-0.186	1.000	-0.127	-0.174	-0.104	-0.114	-0.014	-0.101	0.083	0.418	-0.396
**Actinobacteria**	-0.998	-0.026	-0.127	1.000	0.991	0.998	0.998	0.945	0.698	-0.011	-0.128	0.116
**Bacteroidetes**	-0.991	0.010	-0.174	0.991	1.000	0.990	0.990	0.933	0.692	-0.027	-0.223	0.135
**Firmicutes**	-1.000	-0.020	-0.104	0.998	0.990	1.000	0.999	0.946	0.703	-0.012	-0.120	0.098
**Proteobacteria**	-0.999	-0.011	-0.114	0.998	0.990	0.999	1.000	0.944	0.704	-0.025	-0.130	0.098
**Verrucomicrobia**	-0.945	-0.106	-0.014	0.945	0.933	0.946	0.944	1.000	0.642	-0.006	-0.120	0.050
**TM7**	-0.704	-0.005	-0.101	0.698	0.692	0.703	0.704	0.642	1.000	-0.002	-0.023	0.130
**Euryarchaeota**	0.012	-0.070	0.083	-0.011	-0.027	-0.012	-0.025	-0.006	-0.002	1.000	0.227	-0.398
**Fusobacteria**	0.123	-0.099	0.418	-0.128	-0.223	-0.120	-0.130	-0.120	-0.023	0.227	1.000	-0.202
**Tenericutes**	-0.101	0.079	-0.396	0.116	0.135	0.098	0.098	0.050	0.130	-0.398	-0.202	1.000

Table 2. Correlation matrix.

These additional diagnostics reinforce the limitations acknowledged in the main text, particularly the risk of overfitting due to the number of predictors relative to the number of events. We advise interpreting the regression results with caution and provide these data to ensure full transparency.
